# Supplementary figures and images for: Poor Sensitivity of Fecal Gluten Immunogenic Peptides and Serum Antibodies to Detect Duodenal Mucosal Damage in Celiac Disease Monitoring
Source: Nutrients. 2020 Dec 30;13(1):98. doi: 10.3390/nu13010098 (PMC7824460; doi:10.3390/nu13010098)

# Figure S1

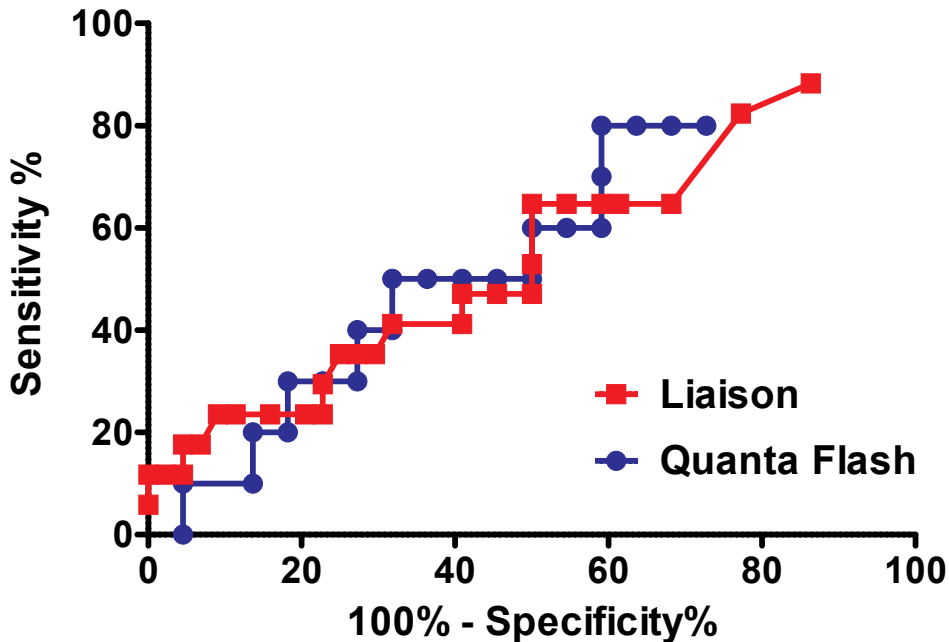

**Figure S2**

**anti-tTG Liaison**

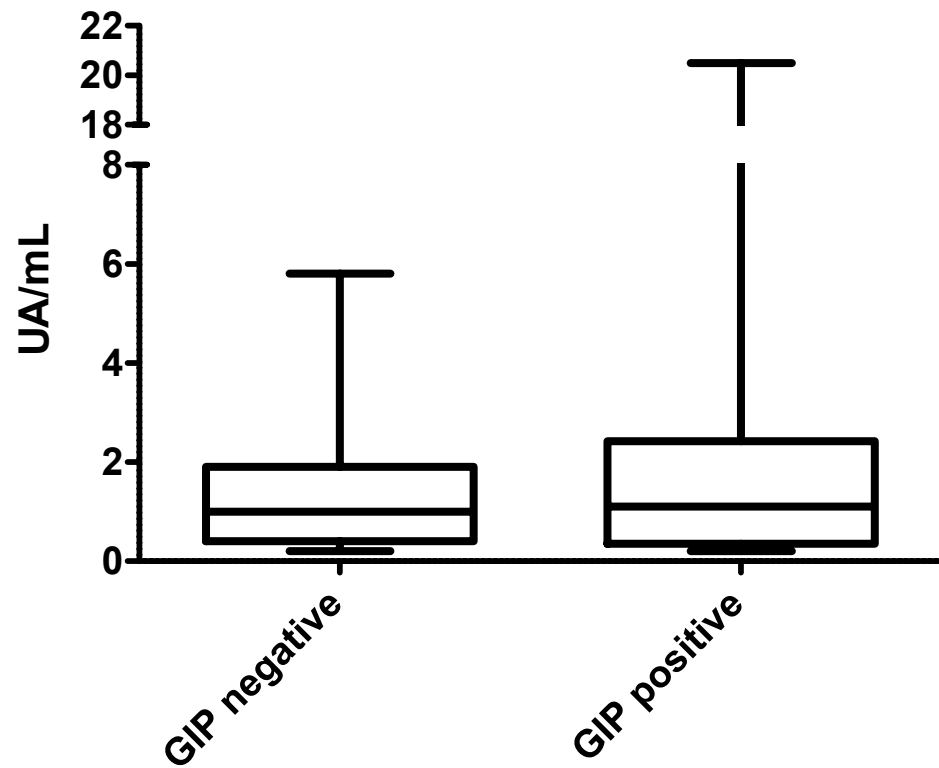

**anti-tTG Quanta Flash**

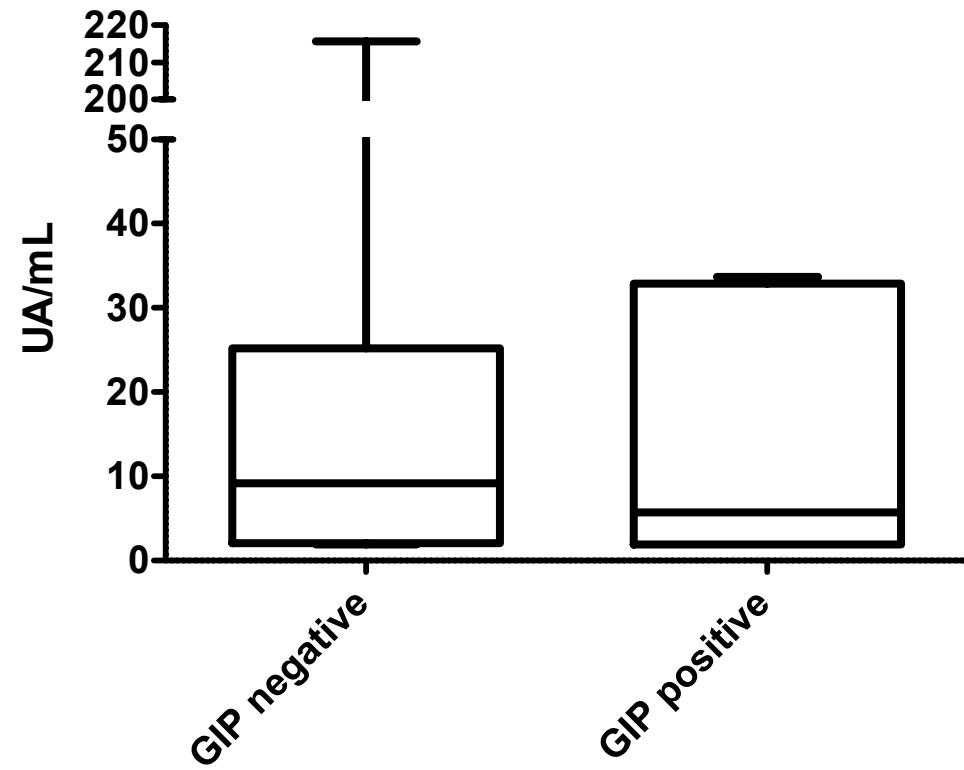

**Figure S3**

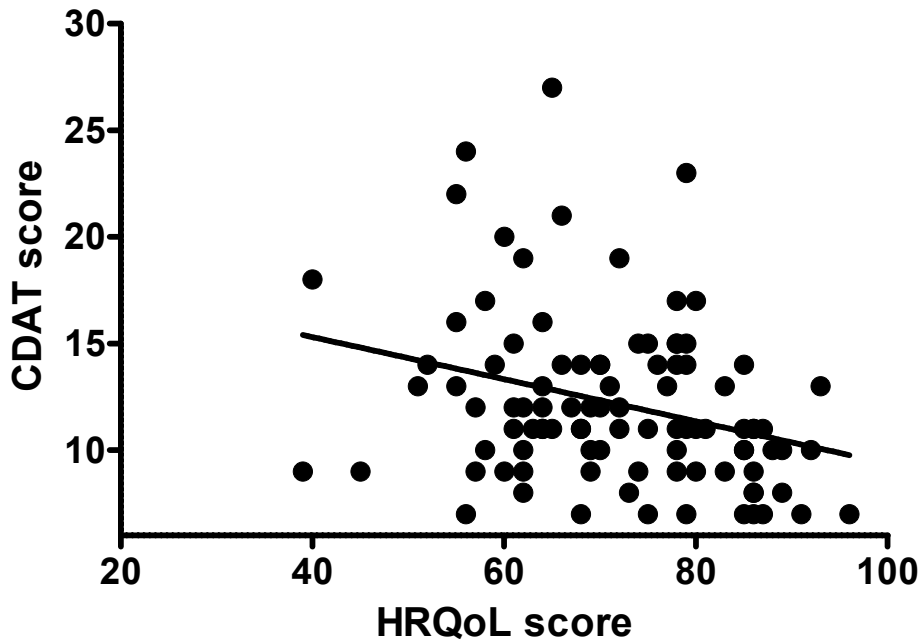

Supplement: Supplementary file 1 [file nutrients-13-00098-s001.pdf]
